# A Dynamic Systems Study on Complexity, Accuracy, and Fluency in English Writing Development by Chinese University Students

**DOI:** 10.3389/fpsyg.2022.787710

**Published:** 2022-05-06

**Authors:** Shuang Zhang, Huiping Zhang, Cun Zhang

**Affiliations:** School of Foreign Languages, Northeast Normal University, Changchun, China

**Keywords:** complexity, accuracy, fluency, development, Complex Dynamic Systems Theory

## Abstract

This study investigated the development of lexical complexity, sentence complexity, accuracy, and fluency in the English writing of 22 Chinese university students from the perspective of Complex Dynamic Systems Theory (CDST). Compositions were assigned 30 times over the course of one academic year through Pigaiwang, an online platform that automatically evaluates writing. A modified retrodictive modeling approach was adopted. Specifically, a longitudinal cluster analysis was used to examine emergent prototypes. A moving correlation analysis and retrodictive interviews were conducted to study the signature dynamics that produce each prototype. At each collection, the 22 student compositions were classified into two clusters. One cluster contained those students who performed better than average in accuracy, but worse in the other three variables. The other cluster comprised those students with the opposite performance. Students moved continuously between the two clusters; and their change trajectories can be categorized into three prototypes: a continuously stable type, an initially variable and then stable type, and a continuously variable type. Case studies of three students representing the three emergent prototypes indicated that the signature dynamics for the three prototypes were related to dynamic interactions among different variables and dynamic changes in affect-related elements in the form of writing interests, motivation, and strategies. The initial conditions and the feedback from Pigaiwang acted as key control parameters in shaping the prototypes. The continuously variable prototype developed their writing proficiency to the greatest extent and had the most variability. Based upon the findings, implications for teaching L2 writing are discussed.

## Introduction

Complexity, accuracy, and fluency (CAF) are the three fundamental dimensions used to measure English learners’ language proficiency (Barrot and Agdeppa, 2021). Research into CAF can reveal interesting information by probing into the multidimensional nature of language use and investigating the cognitive process of language development (Ellis and Barkhuizen, 2005; Polat and Kim, 2014). Traditionally, two different approaches have been widely used in studies on CAF (van Geert and Steenbeek, 2008). One of these approach involves selecting a sufficiently short time span in order to explore the immediate effect of a certain independent variable on CAF, such as task type (e.g., Biber and Gray, 2013; Vasylets et al., 2017; Yang and Kim, 2018; Plakans et al., 2019), working memory (e.g., Gilabert and Muňoz, 2010; Lu, 2015), and so on. Another approach is to measure independent variables over independent subjects. The subjects are treated as representatives of a category in cross-sectional corpus-based studies (e.g., Ai and Lu, 2013; Lu and Ai, 2015). Since the Complexity Dynamic Systems Theory (CDST) was introduced in second language (L2) development (Larsen-Freeman, 1997), L2 systems have been regarded as a complex dynamic process rather than a product. Thus, the two aforementioned methods traditionally used in CAF studies have come under criticism. CDST claims that these approaches oversimplify or overlook complexity, non-linearity, self-organization, complete interconnectedness, and other core characteristics of the language development process (van Geert and Steenbeek, 2008; de Bot and Larsen-Freeman, 2011).

Since Verspoor et al. (2011) offered detailed methods and techniques for the study of complex dynamic systems, CDST has attracted the attention of numerous researchers. CAF research was the first field in L2 development wherein CDST was applied, examining how the behaviors of L2 systems emerge from the interaction of subsystems (e.g., Verspoor et al., 2008; Spoelman and Verspoor, 2010; Verspoor and van Dijk, 2011; Polat and Kim, 2014). In the studies cited above, variabilities received significant attention and were treated as a default function of existence rather than noise (Allen and Boulton, 2011). And it was found that the information contained in inter- and intra- individual variabilities is closely related to the state of system (e.g., Verspoor et al., 2008; Spoelman and Verspoor, 2010). However, the CDST paradigm is also criticized for its lack of generalizable predictions. As some scholars have acknowledged, one of the largest challenges for CDST, which must be addressed, is uncertainty (Morin, 2001; Allen and Boulton, 2011).

How can the accuracy of probabilistic predictions in CDST research be improved? The answer lies in CDST itself. According to CDST, a system is predictable only when it has settled into an attractor state (Dörnyei, 2014), outcome the system prefers to be in Newman (2009) and de Bot and Larsen-Freeman (2011). Therefore, investigating attractor-governed phenomenon is fundamental. As Uprichard (2009) and Dörnyei (2014) suggested, systems’ self-organization characteristics produce several outcomes or types of learners. Identifying the members of each prototype and investigating the signature dynamics of a representative individual is an effective strategy to ensure predictability as members in the attractor-governed prototype would exhibit similar behaviors. It is possible to generalize tendencies, patterns, and contingencies from one system to other similar systems, if they exist under similar conditions. Based on the CDST framework, a retrodictive modeling approach has proposed increasing probabilistic predictions (MacIntyre et al., 2017; Hiver and Al-Hoorie, 2020). Contrary to the usual research making forward-pointing prediction, this approach produces a retrospective model of the development of a system by starting at the end (i.e., the outcomes or types of learners) and then traces back the reasons for ending up with one particular type rather than another (Dörnyei, 2014). Retrodictive modeling approach was applied in studies on individual differences (e.g., Dörnyei, 2014; Chan et al., 2015) and classroom group-work dynamics (e.g., Poupore, 2018), and proved to be useful in making both variability and probabilistic predictions. Unfortunately, it has not yet received much attention in CAF studies. In the present study, we return to the field of CAF and adopt a modified retrodictive approach, with the goal of exploring the group emergence and variability of the CAF of 22 English compositions written by Chinese university students.

## Literature Review

### Complex Dynamic Systems Theory and Retrodictive Modeling

According to CDST, language systems can be considered dynamic systems with several characteristics, including complete interconnectedness, self-organization, adaptability, constant change between attractor state and repellor state^1^, environmental sensitivity, iteration, and so on (de Bot and Larsen-Freeman, 2011, p. 9). Each subsystem develops independently over time and connects with others simultaneously, restricting one another’s freedom (Dörnyei, 2014). Therefore the system is neither “completely random,” nor “wholly predictable” (Larsen-Freeman and Cameron, 2008a, p. 75). The systems’ self-organization characteristics allow it to behave in an orderly manner (de Wolf and Holvoet, 2005) and produce a few outcome patterns. In addition, only when the system is settled into an attractor state is it relatively stable and predictable (Dörnyei, 2014). Governed by strong attractor conglomerates, certain performances are repeated, strengthened, and crystallized. As a result, distinct outcome patterns or learner types emerge, defined as “prototypes” (Dörnyei, 2014, p. 84). More specifically, a prototype’s distinct characteristics are determined and produced by signature dynamics, which are determined by dynamic interactions among the main components of the system. Affect-related factors, external environment factors, and so on may also be driving forces of signature dynamics. In turn, as a repetitive performance, a prototype can be viewed as a kind of attractor state, showing a certain degree of predictability. All members of a single prototype share family resemblance (Chan et al., 2015). Therefore, the study of prototypes, along with their representative individuals can incorporate both group similarities and individual variabilities.

Based on the aforementioned characteristics of a complex dynamic system, Dörnyei (2014) proposed three-step retrodictive qualitative modeling. Starting with the output, the first step is to identify emerging prototypes through interviews and observations. The second step is to determine which members belong to each prototype and find the most representative individual. The final step is to explore the salient components of the representative individual’s system, and the dynamic interactions among those components – or the signature dynamics of the system – that produce distinct outcomes.

This method has been applied to research into individual factors and classroom group-work dynamics (e.g., Chan et al., 2015; Poupore, 2018). However, it has not been applied in research on L2 writing. Based on CDST and our experience teaching L2 English writing, applying retrodictive qualitative modeling to the field of CAF has potential to be relevant and useful.

The present study has modified retrodictive qualitative modeling in the following two aspects. Firstly, to improve the reliability of our research, we utilized longitudinal cluster analysis, which can quantitatively identify prototypes in a bottom-up manner (Peng et al., 2020), rather than identifying prototypes by qualitative methods such as interviews or observation which, despite being relatively subjective, have been common practice in past research (e.g., Dörnyei, 2014; Chan et al., 2015). Secondly, students’ typical trajectories changing between clusters were treated as prototypes herein. In prior research, prototypes were all considered static output, which means each type included students who performed similarly at one point in time. However, researchers have found that learners may not act consistently, but rather change constantly between types or clusters as time goes by (e.g., Dörnyei, 2014; Papi and Teimouri, 2014; Piniel and Csizér, 2015; Han and Hiver, 2018), and there may be regularity in those changes (e.g., Piniel and Csizér, 2015; Han and Hiver, 2018). For example, in a study of the development of L2 motivation, anxiety, and self-efficacy, Piniel and Csizér (2015) found that how students’ trajectories dynamically changed between clusters could also be classified into several types. The unstable nature of L2 development requires that we incorporate this aspect of process into our retrodictive modeling approach. A modified retrodictive modeling approach should increase the accuracy of probabilistic predictions because learners in the same prototype trajectories share similar longitudinal learning patterns (Peng et al., 2020). They belong to the same cluster at almost all the time rather than at one particular time. They also shift clusters at similar points and have a similar degree of variability in their development process.

### Empirical Research on Complexity, Accuracy, and Fluency Based on Complex Dynamic Systems Theory

Until now, CAF studies based on CDST have always focused on an individual’s language development rather than affording the same amount of attention to macro-level group averages as traditional approaches do. Some studies treat CAF as a holistic system (e.g., Larsen-Freeman, 2006; Norris and Ortega, 2009; Verspoor et al., 2012; Polat and Kim, 2014; Li and Sui, 2017; Bai and Ye, 2018; Lowie and Verspoor, 2019; Huang et al., 2021; Verspoor and de Bot, 2021). For example, Larsen-Freeman (2006) investigated the development of grammatical complexity, lexical complexity, accuracy, and fluency of five Chinese EFL learners over 4 months, from the perspective of CDST. The results revealed a high degree of inter- and intra- individual variability in the developmental process. The averaged group data increased in all aspects, but patterns of development for each individual were different from the averaged trajectory. Li and Sui (2017) explored changes in the oral EFL proficiency of six Chinese university students in terms of CAF over the course of 1 year. Sixteen measurements were conducted to analyze multiple dimensions of CAF. Their study found that the development of oral proficiency was not linear. Five kinds of CAF trajectories were identified, including “peaks/valleys,” “ascending in curve,” “descending in curve,” “quasi-horizontal line,” and “mixed mode” (Li and Sui, 2017, p. 392). Meanwhile, other studies have examined how variability drives L2 development (e.g., de Bot et al., 2007; Lowie and Verspoor, 2019; Huang et al., 2021; Verspoor and de Bot, 2021). For example, in research by Lowie and Verspoor (2019), the degree of variability was conceptualized by a coefficient of variation and calculated as standard deviation divided by the mean of the holistic rating score of language proficiency (i.e., complexity, accuracy, and fluency). They found that it was the degree of variability rather than individual difference factors (e.g., motivation) that predicted the final L2 writing proficiency of individual EFL learners.

Other studies have opted to focus on one or two of the dimensions of CAF (e.g., Caspi, 2010; Verspoor and van Dijk, 2011; Baba and Nitta, 2014; Zheng and Feng, 2017; Hou and Chen, 2019; Bulté and Housen, 2020; Evans and Larsen-Freeman, 2020; Yu and Lowie, 2020). For example, Zheng and Feng (2017) traced the development of complexity in 17 Chinese college students. Using dynamic analyses (moving correlations and Monte Carlo Simulations), they found that when the whole system settled into an attractor state, there was no significant interaction between lexical complexity and sentence complexity. However, when the system developed rapidly, the two subsystems began to compete with each other drastically. Bulté and Housen (2020) investigated the dynamic interrelationships between various complexity dimensions in eleven writing tasks by ten Dutch-speaking English learners over 2 years. The results demonstrated that different complexity dimensions sometimes developed in parallel, and at other times became competitive relationships. These studies indicate that L2 writing development is closely related to the interrelationships between subsystems.

Previous studies have documented variabilities both within and across individuals and revealed the dynamic nature of L2 development. However, some gaps in the literature still need to be explored. Firstly, while the variability of an individual’s CAF system has received some attention, the crystallization and emergence of a group system and connections between the whole and individuals have been largely ignored (Zheng, 2020). In order to improve the accuracy of probabilistic predictions, striking a balance between these two focuses is necessary. Secondly, participants in past studies were relatively random and not sufficiently representative. According to Dörnyei (2014), it is important to select individuals that are sufficiently representative of different types of groups, and explore the similarities and differences between those individuals. For example, performing cluster analysis on all the variables in advance is useful for choosing the most representative students. Thirdly, most past research has focused on the dynamic nature of L2 development, while overlooking its complex-adaptive nature. In order to explore how the system interacts with and adapts to the environment, it is necessary to combine a longitudinal quantitative method and a qualitative method to determine the main forces driving phase transition. Such a mixed design renders research more reliable (Hiver and Al-Hoorie, 2020).

To address the aforementioned gaps in the literature and ensure sufficient group predictability and individual variability, this study adopted a modified retrodictive approach to explore the development of CAF in the English writing of 22 Chinese university students. The focus was initially on emergent prototypes, according to students’ typical trajectories changing across clusters. This step yielded insight into the ways CAF typically develops in L2 learners’ writing. Then, one representative student was selected from each prototype to examine their signature dynamics. This analysis revealed the underlying driving forces that produced each prototype and indicated learners’ needs. This study has implications for L2 teachers as it enables them to provide targeted feedback for students of different prototypes.

In sum, previous CAF research based on CDST has demonstrated that inter- and intra- individual variabilities constitute a significant source of insight into the development of CAF learning process, but the emergence of a group system has been largely ignored. In addition, while retrodictive modeling can be considered a useful approach for connecting the whole and individuals, it has failed to capture the unstable nature of L2 development. Therefore, in this study, we modify the retrodictive modeling approach and investigate the prototype trajectories and their underlying signature dynamics, in order to contribute to our better understanding of the developmental features of students’ CAF by striking a balance between the group predictability and individual variability scientifically.

### Research Questions

In order to learn more about the aforementioned aspects, a diachronic corpus was built and a mixed design was created to study the developmental features of CAF in the English writing of 22 Chinese university students. Our two research questions are as follows:

(1) What are the emergent prototypes? Specifically, what CAF profiles can be clustered based on learners’ lexical complexity, sentence complexity, accuracy and fluency? What are the typical trajectories of students moving between these clusters throughout the academic year?
(2) What are the signature dynamics of each prototype? Specifically, what are the dynamic interactions among lexical complexity, sentence complexity, accuracy, and fluency? What are the key sets of influential elements in affect-related and external environmental factors?

## Materials and Methods

In order to investigate CAF in the English writing of 22 Chinese university students, we followed a longitudinal design with data collected 30 times over the course of one academic year.

### Participants

Twenty-two Chinese university students were invited to participate in this longitudinal study. Their names were pseudonyms in this paper. On average they were 18.73 years old. The standard deviation of their ages was 0.88. The youngest student was 18 years old and the oldest, 20 years old; 10 were male and 12 were female. They were all freshmen majoring in English education in the same class at a university in northeastern China. Their average English score on the college entrance examination was 131 out of 150. The lowest score was 118 and the highest was 145. The standard deviation was 6.88, indicating that their initial English language proficiency was relatively homogeneous. They attended 12 h of English class per week, including basic English coursework, grammar, reading English newspapers, a survey of English-speaking countries, and so on. In addition, they were all required to attend an evening class for self-study and homework from 6:30 to 8:30 pm every Monday to Friday. Before beginning data collection, consent was obtained from all the participants.

### Data Collection Procedure

An instructor assigned writing tasks through Pigaiwang once a week during the two semesters of the academic year. Another instructor arranged for the students to complete their first drafts in Pigaiwang independently during their evening class within a limited timeframe. At the end of the academic year, we downloaded 660 drafts written by 22 students, and built the English writing corpus. Retrodictive interviews of representative individuals were also arranged at the end of the year.

### Pigaiwang

The compositions were collected through Pigaiwang^2^, a widely used online platform which can evaluate writing automatically. It has processed 77 million texts over the past 11 years. After students submit their first drafts, Pigaiwang immediately evaluates the composition sentence by sentence and assigns a mark based on its background standard corpus. It not only automatically identifies errors in lexis, grammar, spelling, punctuation and capitalization; but it also offers suggestions on wording or sentence patterns. Students then have the option of revising their writing according to the feedback and can resubmit their compositions as many times as they wish. Students’ submission history and revision process are recorded by Pigaiwang and cannot be deleted.

### Corpus Description

Student writings were collected a total of 30 times over the course of one academic year. It should be clarified that one academic year contained two semesters, each of which lasted 17 weeks. There was a 6-week winter vacation between the two semesters. No writing was assigned over winter vacation. During each semester, a writing task was assigned and collected once a week for the first 15 weeks. Writing was not assigned during the final 2 weeks (week 16 and week 17) because this time period was reserved for revision and final examinations respectively. An instructor assigned writing tasks through Pigaiwang. In order to ensure that students finished the task independently and within a limited timeframe, another instructor arranged for the students to complete their first drafts during their evening class. For each assignment, students were required to write a persuasive essay independently on Pigaiwang with a 200–300 word limit and a time limit of 40 min. Students were not allowed to use dictionaries or reference books when they were writing their first draft and only first drafts were collected in the data analyzed in the present study. All the essay topics were chosen from The International English Language Testing System (IELTS). The selected topics were closely related to students’ daily lives such as *Part-time Jobs*, *Dormitory Security*, *Internet Education*, *Ratings for Teachers*, and so on. At the end of the academic year, 660 drafts written by 22 students were downloaded to build a corpus consisting of 145,450 tokens^3^

### Retrodictive Interview

In order to determine the key driving forces shaping the prototypes, retrodictive interviews of representative individuals of each prototype were arranged at the end of the school year (after 30 writing samples had been collected). These students were asked to recall their English learning experience over the preceding weeks in terms of affect-related factors, including English-writing interest, motivation, and strategies (see Supplementary Appendix I). The retrodictive interviews were conducted on WeChat, which is a widely used social media platform in China. Each interview lasted 15–20 min. The interviews were recorded (audio only) and transcribed for analysis.

### Data Analysis

In the data analysis procedure, first the CAF measuring indexes were checked, and then the raw data was normalized. Longitudinal cluster analysis was used to answer research question 1. Moving correlation analysis and retrodictive interview analysis were utilized to address research question 2. Combining these methods allows them to complement one another and reliably triangulates the writing development process.

### Complexity, Accuracy, and Fluency Measuring Indexes

The indexes for lexical complexity, sentence complexity, accuracy and fluency used in this study are listed in Table 1.

**TABLE 1:** Information on CAF measuring indexes.

	Indexes	Meaning
lexical complexity	low-frequency word types/total word types	the ratio of relatively advanced words in a text
sentence complexity	clauses/T-units (C/T)	the ratio of clauses to T units
accuracy	error free T-units/T-units (EFT/T)	the ratio of error-free T units to total T units
fluency	word/T-units (W/T)	average number of words per T-units

Lexical complexity measures “the proportion of relatively unusual or advanced words in the learners’ text” (Read, 2000, p. 203). It can predict writing quality and the degree of formality (Qin and Wen, 2007; Zhu, 2013). Since unusual or advanced words were conceptualized in terms of word frequency (Laufer and Nation, 1995; González, 2017; Zhang et al., 2021), lexical complexity was measured herein based on Lexical Frequency Profile (LFP) (Laufer and Nation, 1995). LFP consists of four frequency bands, including the first 1,000 most frequent words, the second 1,000 most frequent words, the Academic Word List (AWL) (Coxhead, 2000), and not-in-the-lists words. The last two bands are defined as the low-frequent words (Laufer, 1995). In the present study, lexical complexity was measured by the ratio of low-frequency word types to the total number of types in the text (Liang et al., 2010; Zhang et al., 2021). The Range program (Heatley et al., 2002) was used to automatically calculate the ratio of low-frequency word types in each text by the 22 learners’ and compute lexical complexity thereafter.

Sentence complexity was measured by calculating the ratio of clauses to the total number of T units (C/T) (e.g., Wolfe-Quintero et al., 1998; Wigglesworth and Storch, 2009; Storch, 2009; Crossley and McNamara, 2014; Barrot and Gabinete, 2019). A T-unit is defined as “an independent clause and all its dependent clauses” (Polio, 1997, p. 138). A clause is defined as “an overt subject and a finite verb” (Polio, 1997, p. 139). C/T was used because the production of clause has been shown to be capable of predicting both syntax and wring proficiency (Crossley and McNamara, 2014).

Accuracy was measured by calculating the ratio of error-free T units to the total number of T units (EFT/T) (Wolfe-Quintero et al., 1998; Larsen-Freeman, 2006). For each composition, two highly experienced English instructors identified and coded T-unit boundaries independently and then compared the results. The differences were discussed until an agreement was reached. For error identification, it is reported that Pigaiwang has high reliability and validity in overall error identification (He, 2013), but may make errors when the sentences are long and complex (Bai and Wang, 2019), and this could be compensated by manual verification (Yang and Dai, 2015; Xu et al., 2017). Therefore, in this study, errors were first identified by Pigaiwang and then verified by an instructor. In analyzing accuracy, lexical and grammatical errors were tabulated. Spelling errors were also counted, but those due to inadvertent mistakes in adjacent letter order were not counted for this could be typo problem. For example, if the word “student” was spelled correctly throughout the composition with an exception of “student,” this spelling error was not counted. But they were counted when the student errored more than once. Punctuation and capitalization errors were not counted. Meanwhile, mistakes in word choice were counted only when the word obstruct meaning (Wigglesworth and Storch, 2009; Storch, 2009). This kind of errors were first identified by Pigaiwang, and then verified by two experienced instructors. The differences were discussed until an agreement was reached.

Fluency can be understood more narrowly as a time-related concept, such as the speed of writing (Chenoweth and Hayes, 2001); however the broader sense of fluency is a multidimensional concept, including not only timeliness, but language use ability and content sufficiency as well (Qin and Bi, 2012). Based on these different understandings of fluency, measurement indicators can be divided into two categories: process-based indicators and product-based indicators (Latif, 2009, 2013). The former is a parameter extracted from the writing process considering thinking aloud or observation (e.g., pausing, changes made to the text, or number of words written continuously at a time); while the latter is extracted based on textual features of a writing, including frequency measures (e.g., the total number of words or T-unit) and ratio measures (e.g., the number of words per sentence or T-unit). In the present study, a corpus-based method was adopted and the focus centered on the textual features of each text, therefore a product-based method was necessary (Ma and Qin, 2013). Moreover, in the study by Qin and Bi (2012), ratio measures were found to be more effective than frequency measures in predicting writing quality. Therefore, in this study fluency was measured by the average number of words per T-units (W/T), which has been widely used in past research (e.g., Wolfe-Quintero et al., 1998; Larsen-Freeman, 2006; Storch, 2009; Qin and Bi, 2012; Wang et al., 2015; Xu et al., 2017).

The number of words, clauses, and T-units in the above indexes were all calculated automatically with the help of a web-based L2 sentence complexity analyzer (Lu, 2010, 2011; Ai and Lu, 2013; Lu and Ai, 2015). Compared with manual coding, this tool’s accuracy in structural unit identification is between 0.83 and 1.00 with a reliability of 0.83 to 1.00 (Lu, 2010; Polio and Yoon, 2018).

### Data Normalization

Since the raw data of each indicator was not presented on the same scale, first the data had to be normalized by scaling them between their own minimal and maximal value (between 0 and 1) before conducting longitudinal cluster analysis and moving correlation analysis. In addition, after normalization, it was possible to visualize the developmental trajectories of four subsystems of representative students’ data within the same graph with the help of Excel. In this study, the following formula was utilized: (x-min)/(max-min) (Verspoor and van Dijk, 2011).

### Longitudinal Cluster Analysis

This study used longitudinal cluster analysis with the help of SPSS software to investigate the emergent prototypes. Cluster analysis is an exploratory technique that divides participants into different clusters based on “the similarities between the measurements of variables” (Irie and Ryan, 2015, p. 363). It is useful to portray learner types in “a bottom-up way” (Biber and Staples, 2015, p. 243). Each cluster includes learners who exhibited similar profiles in CAF at one point in time. In order to account for the iterative and longitudinal nature of the data, a longitudinal perspective was adopted. Longitudinal clusters provide fundamental insight into the L2 learning process by investigating how students changed between clusters as time passed. In addition, the frequency of these changes between the clusters can also indicate variability in the development process (Piniel and Csizér, 2015), especially in situations where data collection and cluster analysis are conducted frequently. For example, Piniel and Csizér (2015) used longitudinal cluster analysis (one hieratical clustering and 5 K-means clustering) to examine longitudinal learning patterns in the motivational, affective, and cognitive factors of twenty-one L2 learners. It was found that five prototype trajectories emerged with five corresponding motivation-affect-anxiety relations. The trajectory wherein learners showed high motivation and low anxiety was more variable than another trajectory which scored higher in both motivation and anxiety.

Following the algorithm provided by Piniel and Csizér (2015) and Csizér and Jamieson (2013), for the first composition collection hieratical clustering with Ward’s distance method was used to explore the optimal number of clusters and cluster centers. Lexical complexity, sentence complexity, accuracy, and fluency were the clustering variables and the learners were clustered according to the performance in these four areas. According to the overall cluster solution quality and movement of cluster centers (Han and Hiver, 2018), a 2-cluster solution was finally adopted. The validity of this final cluster solution was checked by canonical discriminant functions with the help of SPSS. Discriminant analysis showed that 100% of the originally grouped cases were correctly classified (*X^2^* = 29.103, *df* = 4, *p* = 0.000), indicating that our 2-cluster solution was quite qualified. For collections two to thirty, K-means clustering was utilized. The initial cluster centers of the four variables utilized for K-means cluster analysis were the final cluster centers from the preceding time. After each clustering, one-way analysis of variance (ANOVAs) was conducted to check the significant differences between the clustered groups. As a result of longitudinal clustering, at each collection point, students with similar CAF performance were grouped into one cluster. Student clusters in collection 1 were linked to student clusters in collection 2, and then to collection 3, and so on (Peng et al., 2020). In this way, longitudinal cluster analysis clearly revealed how the 22 students changed between different clusters as time passed by. Then, students’ change trajectories were classified into several prototypes. Namely, the students who belonged to the same clusters at almost all collection points were grouped into one prototype (Peng et al., 2020), and they had a similar shift points and degrees of variability in development process In this way, longitudinal cluster analysis increased the accuracy of probabilistic predictions, revealed the mechanisms of multicausality and multicomponential L2 systems, and accurately portrayed an individual’s comprehensive development patterns (Peng et al., 2020). The change trajectories were drawn with the help of Excel.

### Moving Correlation Analysis

As mentioned earlier, each distinct prototype was produced by signature dynamics, and the signature dynamics were determined by dynamic interactions among subsystems. Therefore, to answer question 2, one representative student from each prototype was selected and moving correlation analysis was adopted with a window of 5 times (Verspoor and van Dijk, 2011), to investigate how interrelationships among lexical complexity, sentence complexity, accuracy, and fluency changed dynamically over time. Moving correlation analysis is a widely used technique in CDST studies (e.g., Verspoor and van Dijk, 2011; Rosmawati, 2014; Zheng and Feng, 2017; Peng et al., 2020) to visualize the temporal changes in the coefficient values (Caspi, 2010; Verspoor and van Dijk, 2011). Each window overlaps with the preceding one except the first time and correlations can be viewed as a function of time (van Geert and van Dijk, 2002). The first window included the correlation coefficient value in time 1–5, and the coefficient in time 2–6 was then featured in the second window, and so forth. Compared with static correlation analysis, moving correlation analysis is more informative for showing the potential systematic patterns of correlation changes. Programs were written to calculate correlation coefficients with the help of Python project, which is a software for statistical computing (see Supplementary Appendix II). In order to analyze how the interrelationships between subsystems influence the performance of CAF, the development trajectories and trends of the representative students’ CAF systems were also analyzed. Excel was utilized to draw the figures of representative students’ moving correlation among subsystems, developmental trajectories, and developmental trend lines.

### Retrodictive Interview Analysis

As mentioned earlier, affect-related factors and external environmental factors can be driving forces of signature dynamics. Therefore, to answer question 2, semi-structured interviews were conducted to learn about this key set of elements in both affect-related and external environmental factors. After transcribing the interviews, the authors read through the transcripts several times and paid special attention to repeated key words and notable features. Then, interviewees’ initial ideas were extracted and transformed into relevant themes, such as writing interests, writing strategies, and so on. An analysis was undertaken with the goal of discovering any possible connections among different themes.

## Results

This section details the results of the two research questions, including the emerged prototypes and the signature dynamics of each prototype.

### The Prototypes

**FIGURE 1:**
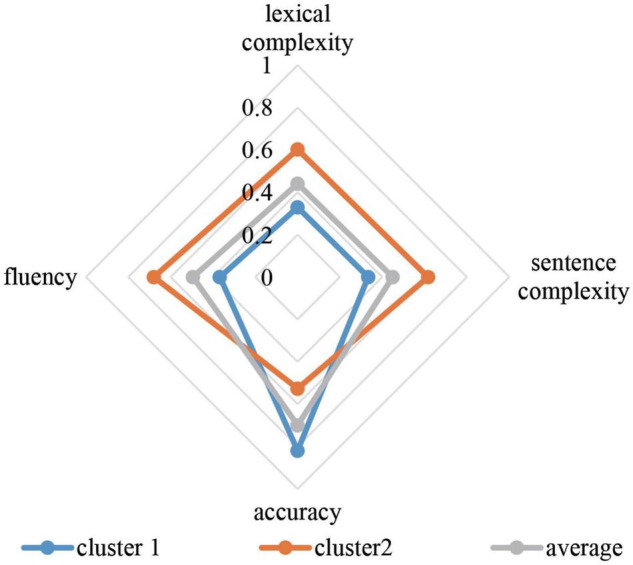
Cluster centers in time 15.

In order to answer research question 1, a longitudinal cluster analysis was conducted. Firstly, the learner clusters at each writing collection were examined. According to the results, the CAF of the 22 students at each writing collection could be grouped into 2 clusters, and the clusters had the same characteristics at each analysis. Let us take a look at a specific example. For time 15 (Figure 1), Cluster 1 includes those students who scored higher than average in accuracy, but lower than average in lexical complexity, sentence complexity, and fluency. On the other hand, the students who performed lower than average in accuracy, but better in lexical complexity, sentence complexity, and fluency fit in Cluster 2. One-way analysis of variance (ANOVAs) showed that there were significant differences between the learners’ CAF performance in the different clusters (see Supplementary Appendix III).

**FIGURE 2:**
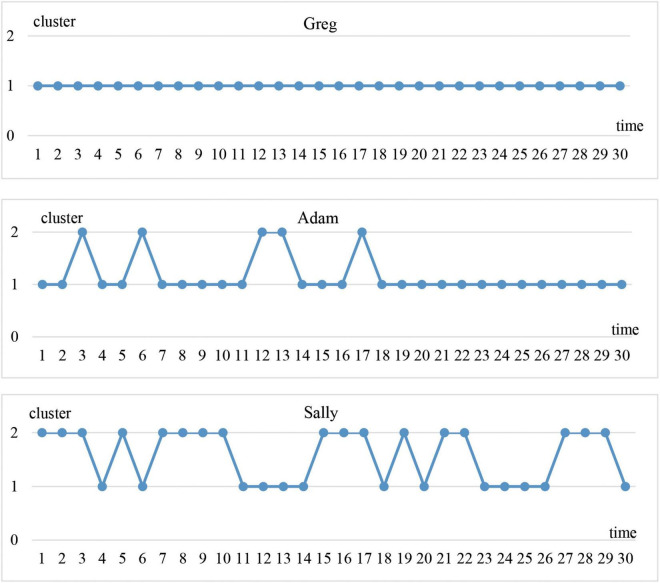
Representative change trajectories between clusters of the three prototypes.

As mentioned earlier, using longitudinal cluster analysis, the student clusters in one time could be linked to the student clusters in the adjacent time. The results reveal that students did not stay in the same cluster during the entire period of reference. They shifted between the two clusters, and their trajectories can be summarized into 3 prototypes (Figure 2).

Prototype 1 is the continuously stable type. There are four students in this type: Danny, Felix, Greg, and Tina. Greg is the most representative one. As shown in Figure 2, Greg stayed stably in cluster 1 in all 30 times over the entire year. The results indicate that students in prototype 1 always performed better in accuracy. They avoided using complex language and seldom made mistakes.

Prototype 2 is the initially variable and then stable type. A total of 9 students (i.e., Adam, Bill, Isabel, Jack, Mary, Quincy, Ruby, Urey, and Vivian) belong to this type, Adam being the most representative. As shown in the trajectory in Figure 2, there were frequent alternations between cluster 1 and cluster 2 from the beginning until time 18, and then it stabilized into Cluster 1. This trajectory shows that students in prototype 2 performed better in lexical complexity, sentence complexity, and fluency but worse in accuracy at the beginning. Then in the middle and later stages, they made fewer mistakes than before and performed better in accuracy than in the other three variables.

Prototype 3 is the continuously variable type. Nine students fall into this category: Carl, Emma, Helen, Kate, Leon, Nancy, Olivia, Paul, and Sally. Sally is the most representative individual. As shown in Figure 2, generally Sally was more variable than Greg and Adam, but there were also some temporary stable periods during the year. For example, after stabilizing in cluster 2 for a while (time 1–3), Sally moved frequently between the two clusters (time 3–7). Then, following a period of movement, Sally settled into cluster 2 again (time 7–10), and then shifted to cluster 1 and settled for a while (time 11–14). It can be inferred that the students in prototypes 3 sometimes performed better in accuracy and at other times performed worse. When these students tried to use more complex language, their accuracy was compromised.

In conclusion, the three prototypes have both differences and similarities. To be specific, in terms of the degree of variability, prototype 1 is the most stable one, followed by prototype 2, and then prototype 3. When the system reached a relatively stable state, all three prototypes preferred to stay in cluster 1. It can be inferred that when students scored higher in accuracy, they settled into the attractor state.

### The Signature Dynamics of the Prototypes: Case Studies

This section deals with the signature dynamics underlying each prototype. Representative students Greg, Adam, and Sally are explored in detail. The moving correlations among the main components in the CAF system and the development of their CAF systems are analyzed. The cluster trajectories in Figure 2 are also taken into consideration. In addition, changes in students’ English writing interests, writing strategies, and the main driving forces in the external environment are explored with the help of retrodictive interviews.

### The Signature Dynamics of Prototype 1: Greg

As mentioned in the previous sections, when the system changed from attractor state to repellor state, accuracy was always compromised. It can be inferred that accuracy is closely related to emergent behaviors of the CAF system. Therefore, it is necessary to focus on the moving correlations between accuracy and the other three variables.

**FIGURE 3:**
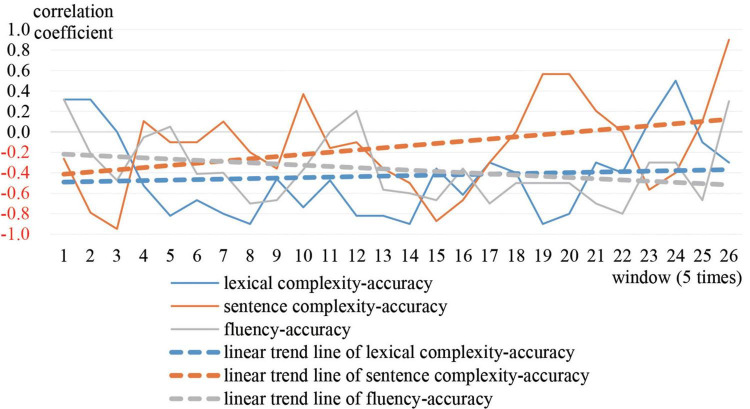
Moving correlations between Greg’s accuracy and other three subsystems.

**FIGURE 4:**
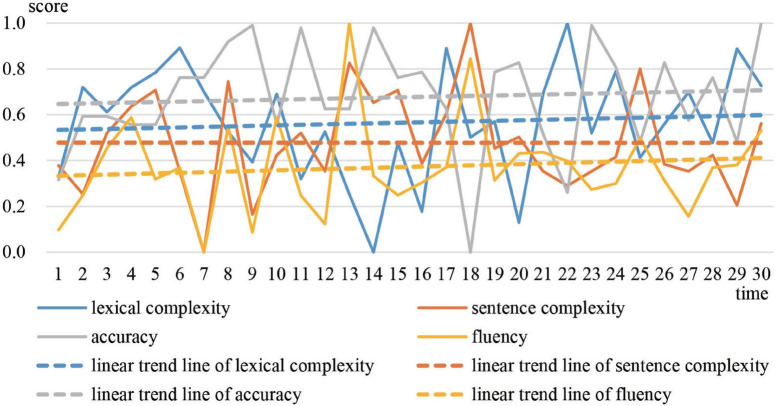
Developmental trajectories and trends of Greg’s CAF.

Greg is the most representative of prototype 1. The moving correlation coefficient between accuracy and the other three variables was calculated respectively. Figure 3 shows the trajectories and linear trend lines of the moving correlations. Figure 4 shows the trajectories and linear trend lines of Greg’s CAF.

As observed in Figure 3, correlations between accuracy and the other three variables were always negative. Although sometimes correlations became positive they soon decreased to negative. The linear trend lines also show that accuracy tended to correlate negatively with lexical complexity and fluency. For sentence complexity, it was not until the end of the year that the correlation between sentence complexity and accuracy increased to a positive level. In addition, as Figure 2 shows, Greg’s CAF system always stayed in cluster 1, which indicates that accuracy tended to win over in the competition with the other three variables.

The linear developmental trends of the four variables in Figure 4 are almost horizontal. This reveals that the overall development of Greg’s CAF was relatively small. The scores on Pigaiwang also confirmed this finding. The scores of Greg’s first two compositions were 79.5 and 80, while the scores of his last compositions were 81.5 and 82.5. Investigation into the corpus shows that Greg’s writings were stagnant in both local language and global structure. He overused some expressions that he had mastered and seldom made errors. For example, “more and more” appeared in the opening paragraphs of 17 compositions to introduce a certain idea related to the various essay topics. Example (1) is an excerpt of Greg’s writing (see Supplementary Appendix IV for the whole text).

(1) It is universally acknowledged that computers are becoming *more and more* popular now. We often use them a lot…as far as I am concerned, I think we should both write with computer keyboards and our hands. (052014011000N.txt).

In the second paragraph, Greg always used “I accept three reasons to explain/interpret that” to introduce his arguments. This phrase appears in 22 writing samples. See the following example (2) (see Supplementary Appendix IV for the whole text):

(2) To illustrate my degree of view, I *accept three reasons to interpret that*. (162014011000N.txt).

In his closing paragraph, Greg tended to use “from what has been mentioned above” to conclude the composition. This expression appeared in 26 of his writings. Here is an example (3) (see Supplementary Appendix IV for the whole text):

(3) *From what has been mentioned above*, on no account can we look down upon these two ideas. (272014011000N).

According to the results of Greg’s interview, it can be observed that Greg was primarily influenced by writing strategies taught in senior middle school. He was taught to use some fixed patterns, frames, or expressions to finish English writing tasks quickly and correctly, and in order to get high marks on the college entrance examination. As he mentioned, “In high school, I have been taught to use some sentences which can be used in all the topics. When I entered college, at the beginning, I’d like to write according to the experience gained in high school.” In terms of writing interest, it seems that he neither likes nor hates English writing, as he said “It’s just an ordinary task that assigned by teacher. Nothing is special.” He also stressed repeatedly that accuracy and clear organization were very important to evaluating the quality of writing. He said that “a high-level writing should first be clear. Then you should use proper vocabularies. It is important to use some authentic vocabularies and sentences. A good writing should be well-organized.” As time went by, he mentioned that he had realized that perhaps he could modify his habits. He said “I think that I’m more skillful than before. I can use some flexible expressions.” However, while he did seem to realize that he could write more flexibly, very few changes can be found in his writing. In addition, as evidenced on the platform Pigaiwang, he seldom revised his writings according to the feedback provided. It was very difficult to effectively alter his initial writing strategies.

In conclusion, it can be inferred that Greg’s signature dynamics are characterized by a long period of a relatively stable state, where accuracy scored higher than other variables. His initial examination-oriented writing strategies strongly influenced his writing performance. Accuracy was dominant over the other three variables.

### The Signature Dynamics of Prototype 2: Adam

Adam is the most representative of prototype 2. The results are as follows.

**FIGURE 5:**
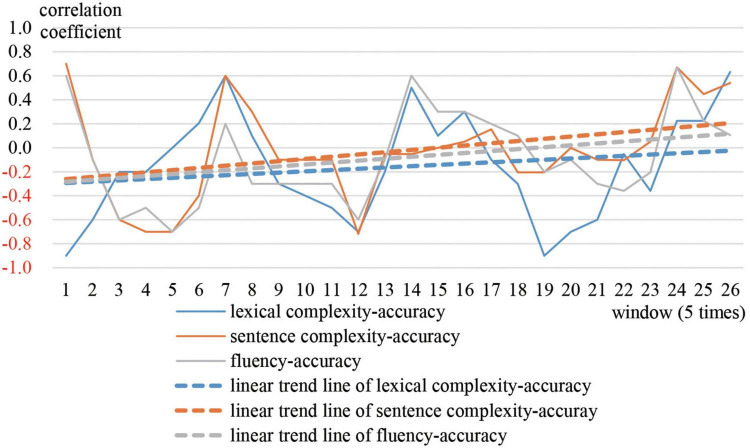
Moving correlations of Adam’s accuracy and other three subsystems.

The trajectories in Figure 5 show that the moving correlation between accuracy and the other three variables fluctuated between positive and negative. To be specific, in terms of correlation between accuracy and sentence complexity, and correlation between accuracy and fluency, the time period spent in negative became increasingly shorter. The value of the correlation coefficient gradually decreased. Regarding correlation between accuracy and lexical complexity, the time spent in negative correlation gradually grew longer, and the value of the correlation coefficient increased. The linear trend lines show that correlations shifted from negative to positive in the middle and end of the academic year.

**FIGURE 6:**
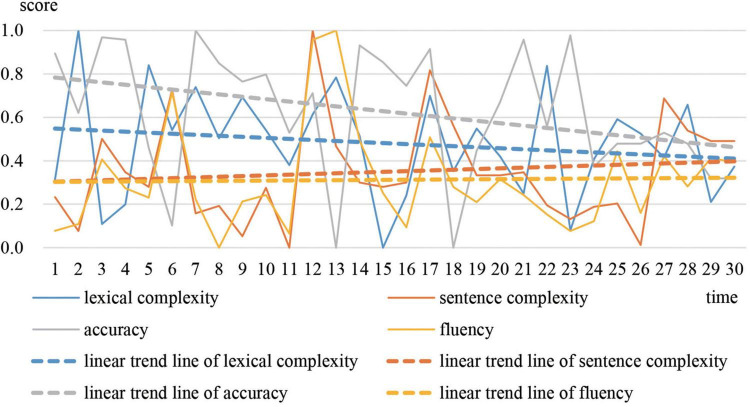
Developmental trajectories and trends of Adam’s CAF.

Figure 6 shows that accuracy decreased the most. Lexical complexity decreased only slightly. Sentence complexity and fluency increased. The gaps between the four variables gradually narrowed. In the end, the normalized values of each variable were around 0.4 and the CAF system turned into an attractor state (cluster 1) where accuracy scored higher. The scores assigned by Pigaiwang for the first two compositions were 75 and 76.5, while the scores of the last two compositions were 82.5 and 83.5. Both the developmental trends of CAF and the scores on Pigaiwang illustrate that Adam made some progress over the year.

The results of the L2 sentence complexity analyzer on the corpus show that the frequency of complex noun phrases decreased, while the frequency of dependent clauses increased. The mean value of the first 15 times of complex noun phrases per T-unit (CN/T) was 1.560, and it decreased to 1.515 in the second half of the year. The mean value of the first 15 times of dependent clauses per T-unit (DC/T) was 0.626, and it increased to 0.707 in the second half of the year. The analysis of the corpus demonstrates that the frequency of object clauses increased. Furthermore, compared to Greg, Adam had fewer repetitive expressions. It can be inferred that Adam tried to utilize complex sentences, especially object clauses in the early stages. At the same time, Adam was not able to pay much attention to lexical complexity and accuracy. After he mastered the object clause, accuracy improved. Then, he no longer attempted to use more complex language, and his writing system shifted into an attractor state. Example (4) and (5) are excerpts of Adam’s writing (see Supplementary Appendix IV for the whole text).

(4) Then, we ought to know *what is important and useful in the books*.

(202014010023N.txt).

(5) But others believe *that schools and teachers are essential for children to learn effectively*.

(272014010023N.txt).

The interview with Adam shows that the feedback from Pigaiwang had a significant influence on his writing strategies and writing interest. He intentionally modified his compositions many times according to the suggestions made by Pigaiwang in order to obtain more satisfactory scores. He made the following comment:

“After receiving the feedbacks from Pigaiwang, I will read the evaluations very carefully in order to know which kind of expressions are recommended as high-level ones. I found that if there are so many simple sentences (without dependent clauses), the scores will be a little low. I’d like to revise the writing for several times, maybe even twenty times, to gain a high mark. Seeing the marks improving from a low score, such as 85, to 88.5, 89, and finally to 90 is such an interesting process!”

After one semester (15 evaluated compositions), he realized that Pigaiwang occasionally made mistakes. He noted:

“I found that Pigaiwang is not almighty. Sometimes it will make mistakes. It will consider a right sentence as a wrong one, especially when a sentence contains several attributive clauses. Maybe it is too complex and too long to make correct judgement.”

According to the aforementioned analysis, some conclusions can clearly be drawn. Adam’s signature dynamics can be understood as movement from a repellor state to an attractor state. In the repellor state, accuracy suffered at the benefit of sentence complexity and fluency as the learner attempted to use complex object clauses. In the attractor state, accuracy was in a supportive relationship with the other variables, because the learner had mastered the object clauses and stopped attempting to use more complex sentences. The feedback from Pigaiwang acted as a critical control parameter shaping Adam’s performance.

### The Signature Dynamics of Prototype 3: Sally

Sally is the most representative of prototype 3. The results are as follows.

**FIGURE 7:**
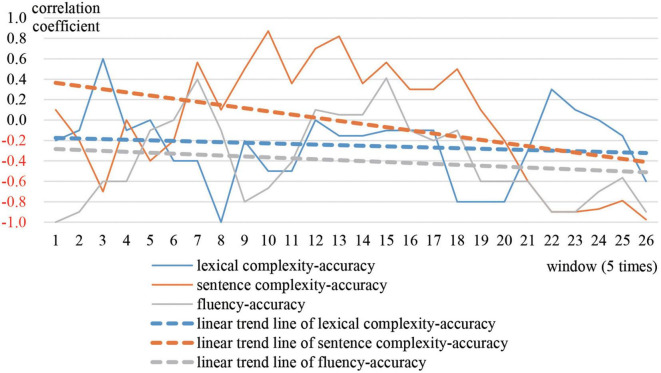
Moving correlations of Sally’s accuracy and other three subsystems.

Figure 7 shows the moving correlations between accuracy and the other three variables. Initially, the correlation between lexical complexity and accuracy fluctuated between positive and negative. The moving correlation coefficient between sentence complexity and accuracy was positive during the middle of the year, and negative at the beginning and end of the year. Accuracy and fluency were positively correlated between time 5–8 and 12–16, and negatively correlated the rest of the time. Combined with the cluster trajectory of Sally in Figure 2, the correlations between accuracy and the other variables were different during each stable period. Thus, it can be inferred that settling into cluster 1 was just superficially stable. The cyclic changes between cluster 1 and cluster 2 constitute another kind of attractor state.

**FIGURE 8:**
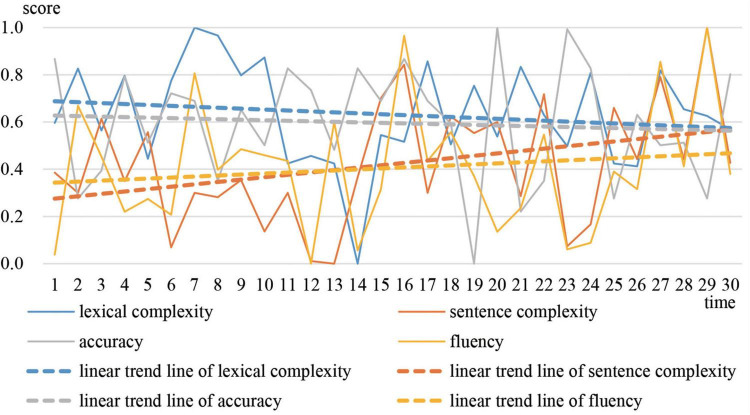
Developmental trajectories and trends of Sally’s CAF.

Sally’s CAF trend in Figure 8 is similar with that of Adam. Lexical complexity and accuracy decreased, while sentence complexity and fluency increased. However, gaps between the four variables continued to narrow and finally the values of the four variables came together at about 0.6, which is larger than the final value of 0.4 of Adam. The scores given by Pigaiwang for the first two compositions were 74.5 and 75, while the last two compositions scored 86 and 85.5. The developmental trends of Sally’s CAF and the composition scores revealed that Sally made greater progress than Adam and Greg.

The results of the L2 sentence complexity analyzer of the corpus show that both complex noun phrases and dependent clauses improved. The mean value of the first 15 times of CN/T and DC/T was 1.804 and 0.75, and in the second semester they increased to 2.158 and 0.989 respectively. When the corpus is analyzed, it is found that in terms of dependent clauses, Sally used increasingly more attributive clauses as time went by.

According to Sally’s interview, the feedback from Pigaiwang motivated her, and she gradually learned to use effective writing strategies during the year. She noted the following:

“At the beginning, I wrote whatever came to mind without organization. Then Pigaiwang will give suggestions not only on languages but also on structure. Gradually I tried to change my writing habits. I learned to write the outlines before writing the drafts. I also tried to use flexible expressions. When I finished the drafts, I often checked and polished the languages by myself before submission. For example, I may change a simple phrase into a more complex sentence.”

She also mentioned that Pigaiwang enhanced her interest in writing. She made the following observation:

“At first, I thought it was a burden to write every week. Very soon, I found that as long as I revised the wrings carefully in the new version, Pigaiwang would give higher marks immediately. I felt a sense of accomplishment seeing the marks getting higher and higher.”

She also volunteered to participate in vocabulary competitions organized by the college. She believed that expanding her vocabulary would also improve her language proficiency to a certain extent.

Based on the above analysis, Sally’s signature dynamics can be described as a cyclical movement between the repellor state (attempting to incorporate more complex language but inevitably making mistakes) and the attractor state (temporarily mastering content learned). Therefore, it can be observed that all variables were actively developing. Competitive and supportive relations were constantly transforming each other, which drove the overall development of CAF.

The signature dynamics of Greg, Adam, and Sally have similarities and differences. During stable periods, the three representative students all performed better in accuracy. However, the correlation between Greg’s accuracy and other variables were competitive, while the correlation between Adam’s accuracy and other variables were supportive. The correlation between Sally’s accuracy and the other variables was different at each stable time. This is because different students focused on different vocabulary and sentence patterns at different stages. In terms of the CAF developmental trends, the four variables of Greg had minimal development. For Adam and Sally, lexical complexity and accuracy decreased, while sentence complexity and fluency increased. But, compared with Adam, Sally’s accuracy and lexical complexity declined to a greater degree, whereas sentence complexity and fluency increased to a greater extent. In terms of overall development, Sally developed the most, followed by Adam, and then Greg. The results reveal that the system with the most variability developed the most.

## Discussion

Based on the aforementioned results, this study demonstrates three emergent prototypes and their underlying signature dynamics. The aforementioned results will be discussed in the following section in relation to the existing literature and CDST.

### The Prototypes

Three prototypes emerged in as students’ CAF developed: a continuously stable type, an initially variable and then stable type, and a continuously variable type. All of the prototypes showed non-linear patterns, which is in line with past research (e.g., Larsen-Freeman, 2006; Li and Sui, 2017; Xu et al., 2017; Bai and Ye, 2018; Bulté and Housen, 2020; Evans and Larsen-Freeman, 2020; Yu and Lowie, 2020). Among the three prototypes, the continuously stable type is similar to the “horizontal line” patterns in Li and Sui’s (2017) research and the developmental trends of the second and third prototypes are similar to the findings of Xu et al. (2017). In addition, when the system changed from attractor state to repellor state, accuracy was always compromised, therefore it can be viewed as the reference variable in longitudinal cluster analysis. This demonstrates that there was trade-off effect between accuracy and other variables. This may be because learners experience a period of trial and error when they attempt to use new expressions (Verspoor et al., 2012). This result coincides with previous findings (e.g., Caspi, 2010; Verspoor et al., 2012; Wang et al., 2015; Xu et al., 2017; Bai and Ye, 2018). For example, in Caspi’s (2010) longitudinal case studies of four L2 learners, the development of lexical and sentence complexity was the precursor of lexical accuracy and sentence accuracy, respectively. Xu et al. (2017) found that accuracy was ignored when learners were attempting to use more complex and fluent language. Our results also show that longitudinal cluster analysis is more adept at distinguishing prototypes rather than simply observing developmental trends (e.g., Bai and Ye, 2018). It can distinguish among students with similar characteristics in developmental trends, but with different characteristics in variabilities according to the frequency and distribution of shift points along the timeline. This type of analysis clearly shows when learners were going through the trial-and-error phases and when they were not.

According to CDST, as a dynamic system, CAF’s interconnectedness, self-organization, and unbalanced internal forces are important factors contributing to the emergence of these three prototypes (de Wolf and Holvoet, 2005; Dörnyei, 2014). Each subsystem restricts the development of the others, reducing the “degrees of freedom” and the “performance that may emerge” (Dörnyei, 2014, p. 85). At the same time, the specific vocabulary and sentence patterns that students focused on, as well as the cognitive resources they distributed, were different. As shown in the previous sections, Adam, Greg and Sally allocated more cognitive resources to accuracy, objective clauses, and attributive clauses, respectively. As a result, certain constructions were constantly strengthened in the process of self-organization and eventually formed prototypes. In addition, this study found that students moved between the two clusters as time passed by. The movements between clusters could also be classified into prototypes. Such relocations and movements mirrored previous findings (e.g., Piniel and Csizér, 2015; Han and Hiver, 2018; Peng et al., 2020) that a complex system underwent phase shifts during its development.

### The Signature Dynamics of the Prototypes

Based on the analysis of moving correlations and developmental trajectories, case studies of three representative students proved that the signature dynamics of the three prototypes were produced by dynamic interactions among different variables. Greg’s accuracy always presented a prominent advantage as compared to the other three variables. The relationship between Adam’s accuracy and the other three variables transformed from competitive to supportive. Regarding the interaction between Sally’s accuracy and the other three variables, there was a closed loop of periodic movement between the competitive and supportive states. This finding demonstrates that it is the dynamic interaction among subsystems from which more complex constructs emerges, which corroborates the results of past research (e.g., Verspoor and van Dijk, 2011; Zheng and Feng, 2017; Bulté and Housen, 2020; Evans and Larsen-Freeman, 2020; Yu and Lowie, 2020).

According to the retrodictive interviews, the signature dynamics of the three prototypes were also related to the dynamic changes of affect-related elements in the form of writing interests, motivation, and strategies. The affect-related elements show two kinds of fundamental forces, namely “approach and avoidance drives” (Chan et al., 2015). To be clear, L2 learners are interested in engaging in situations that bring about positive emotions. For example, Adam and Sally were more interested in and motivated by English writing tasks because they found a sense of accomplishment in seeing their marks improve as soon as they submitted a revised version of their composition. An avoidance drive refer to the tendency to avoid those situations that produce negative emotions. For example, Greg and Adam were afraid of losing points because of errors. When Adam found that complex and long attributive clauses could cause his scores to suffer, he adopted an avoidance strategy. These two kinds of emotional drives underpinned the signature dynamics.

The results also demonstrate the following three characteristics of complex dynamic systems. First, individuals with different initial conditions may perform completely differently, which is in line with the claim of CDST that complex systems are sensitive to their initial state (de Bot, 2008; Lowie et al., 2010; Yu and Lowie, 2020). Yu and Lowie (2020) found that two freshmen with different English experiences in senior high school exhibited distinct degrees of progress after entering college. Similarly, in the present study, since Adam had previously formed some writing strategies, his CAF system was governed by a strong internal attractor state, and it proved challenging for him to change these engrained writing strategies.

Secondly, the feedback from Pigaiwang acted as a key control parameter in shaping prototypes. Positive feedback not only brought about positive emotions in Adam, which is similar to the previous findings (e.g., He, 2013; Shirvan et al., 2020), but it also served to encourage Adam to use more complex sentences adaptively. This corroborates previous findings indicating that a complex system is self-adaptive, in other words, students adjust their cognitive and language resources to perform adaptive language behaviors under different conditions (e.g., Larsen-Freeman, 2006; Evans and Larsen-Freeman, 2020; Fogal, 2020; Chang and Zhang, 2021). According to He (2013), prompt feedback from Pigaiwang can effectively spark students’ writing interest and help students pay more attention to linguistic form. Evans and Larsen-Freeman (2020) found that learners’ adaptation to feedback in linguistic environment motivates learners to use more contextually dominant language.

Thirdly, subsystems do not always compete for limited resources, but actually complement each other at times to reduce the consumption of overall cognitive resources and improve the level of the overall system (Larsen-Freeman and Cameron, 2008b; Vercellotti, 2017, 2019). In terms of the activity of transformation between competition and promotion, Sally was the most active student, followed by Adam, and then Greg. Even if Sally’s accuracy was often at a disadvantage, the comprehensive proficiency of her CAF and scores in Pigaiwang improved more than those of Greg and Adam. In addition, this result also concurs with past findings, i.e., a system with more variability is more likely to develop (e.g., Verspoor et al., 2012; Lowie and Verspoor, 2019; Gui et al., 2021; Huang et al., 2021; Verspoor and de Bot, 2021). For example, Gui et al. (2021) traced the development of 27 Chinese undergraduates’ English-language academic reading ability. They found that the stronger performing students showed relatively more variability over time and used a greater variety of and more sophisticated learning strategies to improve. Curious students who were extremely eager to learn and earn high marks were those who made the most progress. Although this type of student consistently aimed high, because they had not mastered certain skills yet, their results were inconsistent. Nevertheless, such variability is necessary for progress; it is essentially a “symptom” of progress (Gui et al., 2021; Huang et al., 2021).

Finally, let us turn to the question regarding the extent to which the representative students are generalizable to other members within the same prototypes. To our minds, using longitudinal cluster analysis to find prototypes in the first step did fulfill the role of purposive sampling for the subsequent analysis in signature dynamics. It was reassuring to examine the overall improvement and the degree of variability of the average writing score of all the students within each prototype. For overall improvement, the gain score was calculated as the difference between the mean scores of the first two compositions and the last two compositions. For prototype 1, the average scores of the four students for the first two compositions were 77.5 and 78.75 (mean = 78.13), while the last two compositions scored 82.5 and 83 (mean = 82.75). The gain score was 4.62. For prototype 2, the average scores of the nine students for the first two compositions were 76.35 and 76.01 (mean = 76.18), while the scores of the last two compositions were 84.44 and 85.59 (mean = 85.01). The gain score was 8.83. For prototype 3, the average scores of the nine students for the first two compositions were 75.10 and 76.81 (mean = 75.91), while the last two scored 85.94 and 85.61 (mean = 85.78). The gain score was 9.87. Regarding the degree of variability, for each student in each prototype, the coefficient of variation (CV) was calculated as standard deviation divided by the average score of 30 compositions (Lowie and Verspoor, 2019). The mean values of CV for the students in prototype 1 (four students), prototype 2 (nine students), and prototype 3 (nine students) were 0.024, 0.040, and 0.047 respectively. A one-way ANOVA analysis was conducted, and the gain score and CV for each student in each prototype were calculated and entered into ANOVA. The results exhibited significant differences in gain scores among the three prototypes (*F* = 40.707, *df* = 2, *p* = 0.002). LSD *post hoc* tests showed that the gain scores for prototype 3 and prototype 2 were significantly greater than that of prototype 1 (*p* = 0.002; *p* = 0.001). The gain scores for prototype 3 were also greater than that of prototype 2, although they did not attain significance (*p* = 0.645). The results of ANOVA also exhibited significant differences in the CV of the three prototypes (*F* = 18.375, *df* = 2, *p* = 0.000). LSD *post hoc* tests showed that the CV for prototype 3 was significantly greater than that of prototype 2 and prototype 1 (*p* = 0.000; *p* = 0.000), and the CV for prototype 2 was significantly greater than that of prototype 1 (*p* = 0.029). In addition, correlations were calculated between the gain scores and CV for all the 22 students. The results exhibited a strong positive correlation that reached significance (*r* = 0.905, *p* = 0.000). The above analysis reveals that both the overall improvement in writing scores and the degree of variability of prototype 3 were the largest, followed by prototype 2, and then prototype 1, thereby proving that the representative students demonstrated generalizable phenomena to some extent.

## Conclusion

Based on CDST, this research adopted a mixed design to investigate the development of CAF in the English writing of 22 Chinese university students over the course of 1 year. We addressed both group emergence and individual variabilities. A modified retrodictive approach was adopted. Longitudinal cluster analysis was used to investigate the emergent prototypes. The moving correlations, developmental trajectories, and retrodictive interviews were combined to triangulate the signature dynamics which produced each prototype.

Let us turn to the conclusions that can be drawn based on the results of this study. Firstly, at every collection the 22 students’ writings were classified into two clusters. One cluster contains those students who performed better than average in accuracy, but worse in lexical complexity, sentence complexity, and fluency. The other cluster comprises those students with the opposite performance. As students changed between the two clusters, three prototypes emerged: the continuously stable type, the initially variable and then stable type, and the continuously variable type. Secondly, case studies of the three representative students showed that the signature dynamics for the three prototypes were related to dynamic interactions among the different variables, and the dynamic change of affect-related elements in the form of writing interests, motivation, and strategies. Feedback from Pigaiwang also acted as a key control parameter in shaping the prototypes. The continuously variable type developed their writing proficiency to the greatest extent and had the most variability. The overall development of the average writing scores of all the students within each prototype indicates that the representative students demonstrated generalizable phenomena to some extent.

According to the findings of this study, we would like to make some suggestions for foreign language teachers who teach English writing. First of all, teachers should help students (especially students of the continuously stable type) form effective writing strategies and try to reduce students’ dependence on examination-oriented writing strategies. We recommend teachers encourage students to use language flexibly, according to context and purpose, rather than relying upon a limited variety of sentences regardless of context. Secondly, when students attempt to use language that is complex or unfamiliar but appropriate for expressing their ideas, teachers can assign bonus points even if they make mistakes. In other words, teachers should try to encourage students to overcome their fear of making mistakes. In this type of learning atmosphere, students’ L2 writing system would develop in conjunction with variability. Thirdly, our results demonstrate the significant role of feedback in L2 English writing development. For teachers who adopt an online evaluation system, it is necessary to reevaluate feedback from Pigaiwang. When students question the Pigaiwang assessment of certain sentences, teachers should evaluate the composition themselves. And lastly, teachers can select students from the different prototypes to form mixed-prototype groups and organize peer review sessions of English writing tasks. This way, students can learn from one another and share resources.

While we believe that this study has provided some insight into the study of L2 writing development, it should be noted that the CAF indexes in this study do not fully capture L2 writing quality. Thus, more indexes such as the number of non-finite clauses and the number of words written continuously at a time could be incorporated into future research. In addition, larger samples covering longer periods of time would yield a more detailed and comprehensive picture.

## Data Availability Statement

The raw data supporting the conclusion of this article will be made available by the authors, without undue reservation.

## Ethics Statement

The studies involving human participants were reviewed and approved by Professor Committee of School of Foreign Languages, Northeast Normal University. The patients/participants provided their written informed consent to participate in this study.

## Author Contributions

SZ conceived of the initial idea, fine-tuned by HZ. SZ designed the study, collected, and analyzed the data, drafted the manuscript, and finalized the draft for submission. CZ contributed to the manuscript development and editing. HZ revised and proofread the manuscript. All authors have approved the submission.

## Conflict of Interest

The authors declare that the research was conducted in the absence of any commercial or financial relationships that could be construed as a potential conflict of interest.

## Publisher’s Note

All claims expressed in this article are solely those of the authors and do not necessarily represent those of their affiliated organizations, or those of the publisher, the editors and the reviewers. Any product that may be evaluated in this article, or claim that may be made by its manufacturer, is not guaranteed or endorsed by the publisher.

## Footnotes

1 The repellor state is the opposite of attractor state. It refers to the state the system avoids to be in Caspi (2010). To simply put it, there is a “moon’s surface metaphor” of the attractor and repellor state. A ball is rolling over on the surface of the moon with holes and mountains. It will be “attracted” to the holes and “repelled” by the mountains (de Bot and Larsen-Freeman, 2011, p. 15).

2
http://www.pigai.org/

3 “Token” is a term in corpus linguistics which refers to “the total number of words in a text,” while “type” refers to “the number of different words” in a text (Richards and Schmidt, 2010, p. 614).
