# Supplementary material for: A Dynamic Systems Study on Complexity, Accuracy, and Fluency in English Writing Development by Chinese University Students
Source: Front Psychol. 2022 May 6;13:787710. doi: 10.3389/fpsyg.2022.787710 (PMC9120842; doi:10.3389/fpsyg.2022.787710)
Supplement: Supplementary file 1 [file Data_Sheet_1.doc]

**Appendix I Retrospective Interview Guide**

**Interview Questions**

1. From the beginning to the end of the year, how did your attitudes towards writing on Pigaiwang change?

2. From the beginning to the end of the year, how did your writing motivation change?

3. What is a good composition? How did your idea change during the year?

4. From the beginning to the end of the year, do you think your English writing has improved? In which aspects?

5. What writing habits, skills or specific strategies have you developed from the beginning to the end of the year?

6. What influenced your English writing during the year? Are there any influential activities or persons?

**Appendix II Program on Moving Correlation Analysis based on Python software**

import pandas as pd

import numpy as np

import scipy.stats as stats

path='cor.csv'

reader=np.loadtxt(path,dtype=np.float,delimiter=',',encoding='utf-8-sig')

df=pd.read_csv(path,dtype=np.float,delimiter=',',encoding='utf-8-sig')

print(type(df))

r=df.corr(method='spearman')

# raw data

x1=reader[:,0]

x2=reader[:,1]

x3=reader[:,2]

x4=reader[:,3]

#1-4列

for j in range(0,26):

data=pd.DataFrame({'1':x1[j:j+5],

'2':x2[j:j+5],

'3':x3[j:j+5],

'4':x4[j:j+5]})

# print("1组与2组")

# print(data.corr(method='spearman'))

print(stats.spearmanr(data['1'], data['2']))

**Appendix III Descriptive results of the two clusters in Measurement 15**

|  | Group 1(n=13)  Mean (SD) | Group 2(n=9)  Mean (SD) | *F* | *p* |
| --- | --- | --- | --- | --- |
| lexical complexity | 0.329 (0.233) | 0.602 (0.292) | 5.980 | 0.024 |
| sentence complexity | 0.333 (0.207) | 0.616 (0.271) | 7.777 | 0.011 |
| accuracy | 0.819 (0.145) | 0.526 (0.270) | 10.924 | 0.004 |
| fluency | 0.367 (0.167) | 0.678 (0.171) | 18.161 | 0.000 |

**Appendix IV Excerpts from the English Writing Corpus**

**052014011000N**

It is universally acknowledged that computers are becoming more and more popular now, we often use them a lot, including writing them with the computer keyboards. It seems that we write with hands less. As far as I am concerned, I think we should both write with computer keyboards and our hands.

To illustrate my point of view, I have three reasons to explain that. First, Chinese handwriting is a symbol of our traditional culture. Our Chinese people should keep the good writing as our duties. Second, the society has changed a lot these years, writing by keyboards is gradually becoming a common way to deal with things. We are able to practice our handwriting skills on computers, too. Only by doing these can we meet the demand of society. Finally, both of ways will benefit us a lot. We can write with hands to finish applications and compositions, etc. We also write with keyboards to accomplish online homework and chatting with our friends. Thus it is of great significance to practice writing by these two ways.

From what has been mentioned above, we can not deny the fact that both of writing ways are convenient for us to be adapt to morden society. We are not able to give up either of them. Only if practice in both ways can we enjoy ourselves in Chinese handwriting better.

**162014011000N**

Nowadays there are more and more schools asking their students to give grades to their teachers. Opinions vary from person to person. Some people explain that the idea is trashy, but others think it is an useful way to test both students and teachers. As far as I am concerned, I agree with students' rating of their teachers.

To illustrate my degree of view, I accept three reasons to interpret that. First, this is a good way which can report the results of teaching. Schools can judge teachers' working effectiveness an efficacy. Second, this is an effective way that can test the feelings between teachers and students. If their relationship is perishing, thus a better teaching way should be adopted to solve the problems. Finally, teachers are one of the staff in schools. Like waiters, they should also need to be rated. If they work worse, they accept the reasons to be dismissed. Only if by this way can teachers work better and better.

From what has been mentioned above, we can draw an ending that not only students but also teachers should continually promote themselves in order to meet the demand of society. Those behave well will gain a lot. Thus I support students' rating of their teachers.

**272014011000N**

It is universally acknowledged that the Internet has widely spread all over the world. The development of Internet has become one of the ways for children to study. Opinions about this vary from person to person.

Some supporters say that computers and the Internet are more important for a child's education than going to school. Children become clever than old days. At the same time the Internet is convenient. Children can accept knowledge on their own. There are lots of useful information on the Internet, which can broad kids' horizon. Thus there's no need to go to school. It is an innovation in educational system.

Nevertheless, objectors argue that schools and teachers are essential for children to learn effectively. Chinese educational system has accepted this way for many years. Children should attend school not only to gain knowledge, but also to communicate with others. In school children can strengthen both academic and social skils. In a word, students can not miss school's education.

As far as I am concerned, I recommend that education can combine these two methods. There is no doubt that the Internet can bring much convenience, but bad news often occur on the Internet which can leave children negative effects. Also in school there aren't plenty of resources students can use. But the Internet can provide resources a lot.

From what has been mentioned above, on no account can we look down upon these two ideas. Only if by connecting them can children get good education.

**202014010023N**

As many people believe, reading is a critical part of learning and growth, but others think learning best from first-hand and concreting experience, using our senses, exploring our environment of people, things, places and events. In my opinion, I think they are both important.

First, we need to read, and then we can have enough knowledge to experience, but we should be constantly improved in the practical exploration and give a big step forward to knowledge is from books, so we need to learn how to choose a good book. Then, we ought to know what is important and useful in the books. For practicing, I believe we need to seizing the opportunities as much as possible, and we should not be shy when we participate a society practice.

I think as far as we learn well and practice more, we must be able to a successful man, finally.

**272014010023N**

Recently, there is one sound saying that computer and the Inernet are more important for a child's education than going to school. But others believe that schools and teachers are essential for children to learn effectively.

Of course, in my opinion, I think school is much more importaant, especially for those children whose minds are not mature enough. Therefore, they need a lot help from their teachers at school. For another, as we all know, parents are always busy in their business to support the family. In this case, teachers play the most important roles in taking care of children and helping them grow up. May you say there still are some examples that some adults who are successful didn's get enough school education when they were young. But you have to know tnat a man can be a success doesn't depend on how much knowledge he got before. It all relies on whether he is acivilized human. So those people must get enough ideological education from all the aspects, and that is what the Internet and computer cannot bring to us.

In addition, going to school is not only help your grow up, but also bring you many valuable things, like friendship, value and so on. In contrast, if you are always addicted in the Internet and computer, may you get some online friends, but how can you make sure they are true friends?

To sum up, we can see going to school has too many advantages. However, attaching importance to school education doesn't mean ignoring the Internet and computer. Moreover, it is the age of information now and imformation is very important in the society. We can't yield ourselves up to be a web illiterate. Therefore, I suggest while in study courses, please insist on getting network knowledge.
